# Molecular Mechanisms of Gut Microbiota–Immune System Crosstalk: From Mucosal Architecture to Adaptive Immunity Programming

**DOI:** 10.3390/ijms27146246

**Published:** 2026-07-14

**Authors:** Dana Ciaușu-Sliwa, Robert Capotă, Andra-Cristina Bostănaru-Iliescu, Valentin Năstasă, Mihai Mareș

**Affiliations:** Faculty of Veterinary Medicine, “Ion Ionescu de la Brad” Iasi University of Life Sciences, 700490 Iasi, Romania; dana.ciausu@iuls.ro (D.C.-S.); robert.capota@iuls.ro (R.C.); andra.iliescu@iuls.ro (A.-C.B.-I.); valentin.nastasa@iuls.ro (V.N.)

**Keywords:** gut microbiota, immunometabolism, adaptive immunity, short-chain fatty acids, bile acid signaling, aryl hydrocarbon receptor, MR1, regulatory T cells, dysbiosis, microbiota-based therapy

## Abstract

The mammalian gut microbiome functions as a metabolically active immunological organ and has co-evolved with its host to maintain systemic homeostasis. This review integrates current evidence on the molecular mechanisms governing bidirectional microbiota–immune communication, emphasizing evolutionary conservation, receptor-mediated signaling, and translational implications. Microbial structural ligands and metabolites—including short-chain fatty acids, bile-acid derivatives, and tryptophan catabolites—engage host receptors such as G-protein-coupled receptors, FXR/TGR5, and the aryl hydrocarbon receptor (AhR), thereby regulating epithelial barrier integrity, regulatory T-cell differentiation, Th17 polarization, mucosal IgA production, and systemic immune tone. Riboflavin-derived metabolites presented via major histocompatibility complex class-I-related molecule (MR1) further shape mucosal-associated invariant T-cell development (MAIT), illustrating metabolite-driven immune system programming. Dysbiosis induced by antibiotics, dietary perturbation, or aging disrupts these molecular networks, promoting chronic inflammatory, metabolic, autoimmune, and neuroimmune disorders. Comparative analyses across mammalian systems underscore conserved pathways of host–microbe coadaptation and immune education. Therapeutically, microbiota-modulating strategies—including probiotics, prebiotics, synbiotics, fecal microbiota transplantation (FMT), postbiotics, and IgY-based passive immunotherapy—aim to restore immunometabolic signaling. Emerging in vitro and in silico platforms further provide mechanistic precision while supporting ethically aligned translational research. Collectively, these insights position microbiota-derived molecular signaling as a central determinant of adaptive immune architecture and a targetable axis in precision immunotherapy.

## 1. Introduction

In recent years, the gut microbiome has emerged as a key determinant of host health, with effects that extend well beyond digestion. The mammalian gastrointestinal tract hosts a diverse, dynamic microbial community that maintains a mutualistic relationship with the host. A central feature of this relationship is its bidirectional communication with the immune system: the gut microbiota supports immune development and maturation, while immune responses continuously shape microbial composition throughout life.

The mammalian gastrointestinal tract harbors a vast and complex consortium of microorganisms, collectively referred to as the gut microbiota [1]. This community is composed of bacteria but also includes viruses, fungi, and archaea. It fulfills essential physiological functions that contribute significantly to host health. Notably, bacterial species within the gut microbiome metabolize undigested dietary components and host-inaccessible substrates, leading to the production of a wide array of diet-derived and secondary metabolites, collectively known as the metabolome [2].

Long before the advent of next-generation sequencing (NGS) technologies which enabled precise characterization of the gut microbiota, traditional Chinese medicine recognized the therapeutic relevance of fecal material. Over 1700 years ago, ancient Chinese physicians utilized preparations such as fresh fecal suspensions or dried feces—referred to as yellow soup or Huang-Long decoction—to manage cases of severe diarrhea and food poisoning [3].

Growing evidence suggests that microbial metabolites, surface structures, and microbe-associated molecular patterns (MAMPs) play pivotal roles in regulating immune tolerance, inflammation, and barrier integrity. Comparative studies of germ-free animals and conventionally raised laboratory animals have highlighted a symbiotic relationship between the host and its gut microbiota. Conversely, the immune system shapes the microbial landscape through secretory immunoglobulins, antimicrobial peptides, and cytokine-mediated responses [4].

With advancements in life sciences and molecular techniques, the study of the gut microbiome has become a focal point in biomedical research. It is now well established that disruptions or imbalances in gut microbial composition and function—collectively termed dysbiosis—are associated with a broad spectrum of human diseases, ranging from gastrointestinal disorders and obesity to cardiovascular diseases, allergic and autoimmune conditions, and neurological pathologies [5,6,7]. However, a fundamental question remains unresolved: do these microbial alterations serve as a cause or a consequence of disease?

This review examines the molecular mechanisms that mediate communication between the gut microbiota and the host immune system, with particular emphasis on microbial metabolites, extracellular vesicles, receptor-mediated signaling pathways, and their role in adaptive immune programming. Unlike previous reviews that focus primarily on microbiota composition, mucosal immunology, or microbiota-associated diseases separately, this review integrates structural, microbial, and immunological perspectives within a unified mechanistic framework linking microbial-derived signals to local and systemic immune regulation.

The following sections examine the structural, microbial, and immunological foundations of host–microbe interactions before discussing the molecular mechanisms that govern immune regulation and adaptive immune programming.

## 2. Literature Search Strategy

This manuscript was prepared as a narrative review aimed to synthesize current knowledge on the molecular mechanisms underlying gut microbiota–immune system crosstalk and adaptative immune programming. The review focuses on the interactions between microbial communities, microbial-derived signaling molecules, epithelial barriers, and host immune responses, with particular emphasis on receptor mediated pathways and translational implications.

Relevant literature was identified through searches of PubMed, Scopus, and Web of Sciences databases conducted between October 2025 and May 2026. The search strategy included combinations of the following keywords and related terms: “gut microbiota”, “microbiome”, “immune system”, “mucosal immunity”, “adaptive immunity”, “host-microbe interactions”, “gut-brain axis”, “gut-immune axis”, “extracellular vesicles”, “microbial metabolites”, “regulatory T cells”, “Th17 cells”, “MAIT cells”.

Original research articles, clinical studies, systematic reviews, and meta-analyses were considered based on their relevance to microbiota–immune system interactions and associated molecular mechanisms. Earlier studies were considered when necessary to provide historical context and support key concepts. Additional references were identified through manual screening of relevant publications.

As a narrative review, this work was not conducted according to systematic-review methodologies and did not aim to provide an exhaustive quantitative assessment of all available evidence. Instead, the objective was to integrate current experimental, clinical, and translational findings into a coherent mechanistic framework describing the role of the gut in immune regulation and adaptive immune programming.

## 3. Functional Morphology of the Gastrointestinal Tract of Humans and Commonly Used Laboratory Animals

Together with the respiratory, cardiovascular, and excretory systems, the digestive system supports the body’s nutritional needs. Understanding the functional morphology of the gastrointestinal tract (GIT) across species provides foundational insights into host–microbiome–immune interactions and guides model selection in translational research [8].

**Figure 1:**
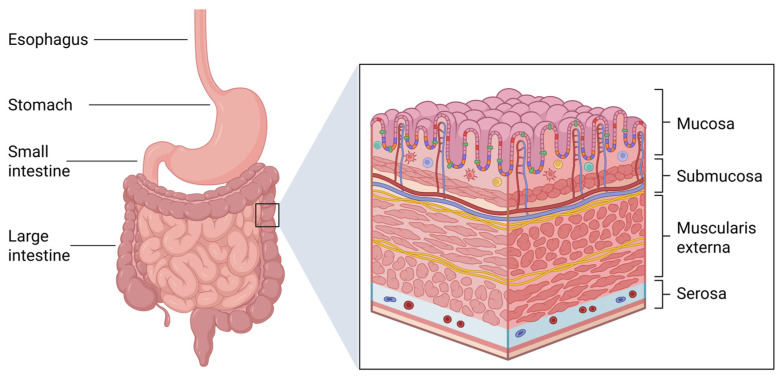
General morphology of the mammalian gastrointestinal tract (Created with BioRender.com). Schematic representation of the gastrointestinal tract showing the major segments (esophagus, stomach, small intestine, large intestine) and the structural organization of the GI wall (mucosa, submucosa, muscularis externa, serosa).

The GI wall comprises four layers—mucosa, submucosa, muscularis externa, and serosa—along the oral cavity, esophagus, stomach, small intestine, and large intestine (Figure 1). Despite this conserved architecture, each segment exhibits structural and functional specializations that shape microbial colonization and immune activity. The mucus layer and epithelium vary in thickness and goblet cell density across regions and species, while epithelial turnover remains rapid in all models.

The epithelium is composed of multiple specialized cell types, each contributing to digestive and immune functions. In the stomach, the epithelium contains parietal cells (secreting hydrochloric acid), chief cells (producing pepsinogen), and mucous neck cells (secreting protective mucus), whereas the intestinal epithelium comprises enterocytes, enteroendocrine cells (EECs), goblet cells, tuft cells, Paneth cells, and the intestinal stem cells [9,10].

Together, these cell populations maintain nutrient absorption, barrier integrity, antimicrobial defense, and host–microbiota communication [10,11,12,13]. This dynamic epithelial compartment is continuously renewed by proliferating Lgr5+ stem cells located at the base of the intestinal crypts, which generate differentiated cell lineages that migrate along the crypt–villus axis and replace surface epithelial cells within a few days [9,10].

**Figure 2:**
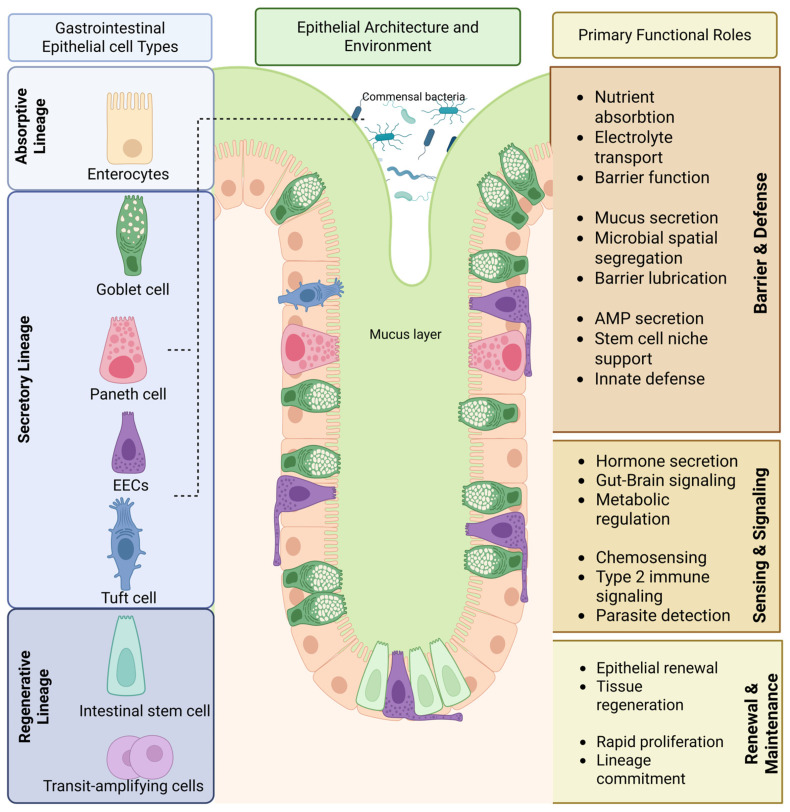
Gastrointestinal epithelial cell types and their functional roles (Created with BioRender.com). The schematic illustrates the major epithelial cell populations of the gastrointestinal tract, including enterocytes, goblet cells, Paneth cells, enteroendocrine cells, tuft cells, and intestinal stem cells, together with their principal functions in nutrient absorption, mucus production, antimicrobial defense, epithelial regeneration, and microbiota–immune system interactions.

Figure 2 summarizes the major epithelial cell populations of the gastrointestinal tract, highlighting their secretory products, primary physiological functions, and their roles in shaping host–microbiota–immune system interactions.

Enterocytes are the most abundant intestinal epithelial cells and primarily absorb nutrients, electrolytes, and vitamins. Beyond this absorptive role, they function as immune sentinels by expressing pattern recognition receptors (PRRs) which detect microbial-associated molecular patterns. Enterocytes also release cytokines and chemokines that shape local immune responses and mediate communication with underlying dendritic cells (DCs) and macrophages. Microbial metabolites, especially short-chain fatty acids (SCFAs) such as acetate, propionate, and butyrate, directly influence enterocyte function. Together, these functions position enterocytes as a key interface through which microbial metabolism influences host immune and metabolic responses.

Goblet cells secrete mucins, mostly MUC2, which form the mucus layers covering the intestinal epithelium. This mucus barrier provides a physical and biochemical interface that both limits microbial access to epithelial cells and offers a niche for commensal microbes [14]. The composition and thickness of the mucus layer vary across intestinal segments and between species, influencing microbial colonization patterns. Dysbiosis or impaired goblet cell function reduces mucin secretion, leading to a thinner, discontinuous mucus layer that allows pathogens to reach the epithelial surface and trigger inflammation. Conversely, signals from commensal bacteria stimulate goblet cell differentiation and mucin production, establishing a feedback loop that maintains mucosal homeostasis [15]. Alterations in goblet cell activity are linked to inflammatory bowel diseases, highlighting their central role in microbiota–immune system equilibrium [16,17].

Paneth cells are specialized secretory epithelial cells that produce antimicrobial peptides (AMPs), including α-defensins, lysozyme, and RegIIIγ, located at the base of intestinal crypts [18,19]. These molecules directly control microbial populations and prevent bacterial overgrowth near the stem cell niche. Paneth cell function is influenced by microbiota: microbial signals via PRRs stimulate AMP production, while SCFAs and other metabolites regulate their secretory activity. In addition, Paneth cells secrete growth factors such as Wnt3, EGF, and Notch ligands, which are critical for maintaining the adjacent intestinal stem cells [20]. Dysregulated Paneth cell activity contributes to intestinal dysbiosis, impaired epithelial renewal, and inflammatory conditions such as Crohn’s disease [18,21,22,23]. Thus, Paneth cells serve a dual function: antimicrobial defense and regulation of epithelial regeneration [23].

Although they comprise only ~1% of epithelial cells, EECs are essential regulators of gut physiology and host–microbiota communication. Distributed throughout the gastrointestinal tract, they represent the largest endocrine system of the human body, secreting peptide hormones such as glucagon-like peptide 1 (GLP-1), peptide YY (PYY), and serotonin (5-HT), which coordinate local digestive processes, regulate appetite and glucose metabolism, and influence systemic energy homeostasis [24,25]. Recent evidence highlights that microbial metabolites stimulate EEC hormone release, linking the microbiota to gut–brain signaling and metabolic control [26]. Through this axis, EECs integrate luminal microbial cues with neural and endocrine pathways, contributing to both host metabolism and immune regulation.

Intestinal stem cell activity is tightly regulated by Wnt, Notch, and BMP signaling pathways, which are influenced by microbiota-derived metabolite [27]. In addition, Paneth cell-derived Wnt ligands and microbial cues together maintain stem cell niche integrity. Disruption of these signals by dysbiosis or infection leads to impaired regeneration, compromised barrier function, and heightened susceptibility to inflammation [20]. Thus, intestinal stem cells represent a critical point where the microbiota influences epithelial turnover and long-term tissue homeostasis [28]. Recently, tuft cells have emerged as important chemosensory epithelial regulators that contribute to microbiota–immune system crosstalk by sensing luminal signals and initiating type 2 immune responses through IL-25-mediated activation of innate lymphoid cells [29].

Understanding the functional specialization of gastrointestinal epithelial cells and their interactions with the gut microbiota provides an essential cellular framework for investigating host–microbe–immune system dynamics. However, the mechanistic study of these interactions in humans remains constrained by ethical and experimental limitations. To overcome this, a range of animal models has been employed to replicate key aspects of gut physiology, microbiota composition, and immune system development. Ranging from simple invertebrates such as *Hydra* [30,31] and Annelids [32,33] to more complex vertebrates like mice and rats, each organism contributes distinct methodological strengths tailored to specific scientific inquiries. Rodent models offer robust genetic manipulability and the capacity for engraftment of entire human-derived microbial communities, facilitating translational research [34,35,36,37,38,39,40,41,42,43,44,45]. In contrast, non-mammalian models provide valuable insights into evolutionarily conserved host–microbe interactions and fundamental metabolic processes [46,47,48,49]. As microbiome research continues to evolve, the strategic selection of appropriate animal models, alongside the integration of advanced analytical technologies, will remain essential for elucidating the multifaceted roles of the gut microbiota in health and disease.

Table 1 compares gastrointestinal structural and immunological features across humans, mammalian models, and selected non-mammalian models. Rodents and pigs provide the strongest translational relevance, whereas zebrafish enable real-time visualization of microbial colonization and immune responses in a vertebrate system [50]. *Drosophila*, though lacking adaptive immunity, offers exceptional genetic tools to study conserved innate immune pathways and host–microbiota interactions [16,51,52,53,54,55].

The structural morphology of the GIT plays a pivotal role in shaping host–microbe interactions, particularly considering growing evidence linking gut microbial communities to host health. However, inter-individual variability in GIT architecture remains insufficiently characterized [61], limiting our understanding of the underlying causes of this variation and its consequences for microbial composition and function. Model selection should align morphological and immunological traits with research aims—rodents for mechanistic and germ-free studies, and pigs for translational outcomes.

In summary, the gastrointestinal tract displays a conserved layered architecture across species, yet with notable differences in epithelial specialization, mucus barrier organization, and distribution of gut-associated lymphoid tissue. These structural variations influence microbial colonization patterns and immune responses, thereby shaping the outcome of host–microbiota interactions. Collectively, they provide the anatomical foundation for the molecular mechanisms of microbiota–immune system crosstalk discussed in the following sections.

## 4. The Composition and Function of the Gut Microbiome in Mammals

The mammalian gastrointestinal tract harbors a vast and dynamic microbial ecosystem whose structure closely reflects host anatomy, diet, and physiology. This consortium, composed primarily of bacteria but also archaea, fungi, and viruses, performs essential metabolic and immunological functions that have co-evolved with their hosts. Comparative immunological analyses demonstrate that this co-evolution shaped both microbial symbiosis and the architecture of mammalian immune systems, with conserved mechanisms of mucosal tolerance and immune regulation arising alongside microbial diversification [56]. Although the exact taxonomic composition differs among species and individuals, a conserved functional repertoire persists across mammals, underscoring the evolutionary interdependence between microbes and their hosts [1,50].

Across mammalian species, the gut microbiota is dominated by two phyla—*Firmicutes* and *Bacteroidetes*—which together usually account for more than 80% of total bacterial abundance [6,7]. Within these groups, genera such as *Clostridium*, *Lactobacillus*, *Ruminococcus*, *Faecalibacterium*, and *Bacteroides* represent key functional players involved in fermentation and host–microbe communication. Other taxa—*Akkermansia muciniphila* (*Verrucomicrobia*) and *Bifidobacterium* spp. *(Actinobacteria)*—occur at lower abundance yet play essential roles in mucin degradation and immune system maturation, respectively [26,62].

**Figure 3:**
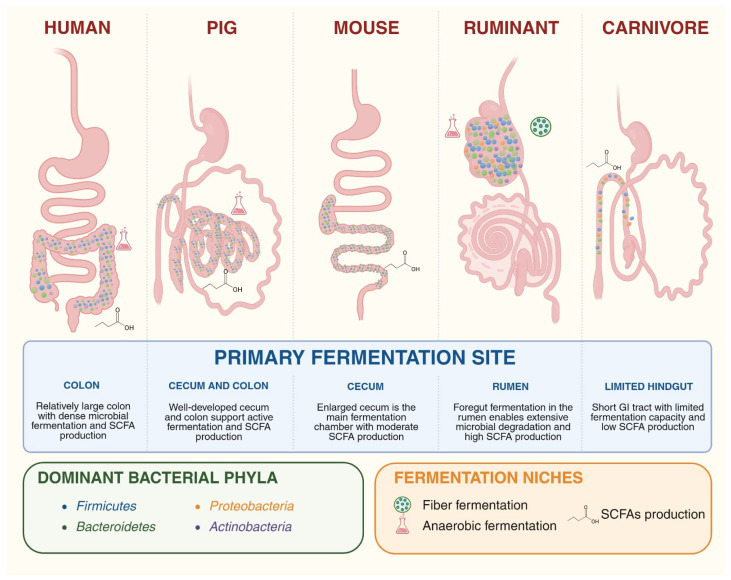
Comparative organization of the mammalian gastrointestinal microbiome across representative species (Created with BioRender.com). Schematic overview of the gastrointestinal tract of human, mouse, pig, ruminant, and carnivore hosts illustrating dominant bacterial phyla and their qualitative distribution along anatomical compartments. Icons indicate principal fermentation niches (rumen, caecum, colon) across species.

Microbial density and diversity increase progressively from the stomach to the colon, paralleling gradients in pH, oxygen, and nutrient availability. The stomach and proximal small intestine host relatively sparse microbial populations due to acidity, bile, and digestive enzymes, while the distal ileum, caecum, and colon form anaerobic environments supporting densities exceeding 10^11^–10^12^ cells per gram of content [61]. In these niches, obligate anaerobes dominate, particularly those capable of fermenting complex carbohydrates and host-derived mucins. Comparative gastrointestinal anatomy underlies host-specific microbiome architecture (Figure 3). Herbivorous and omnivorous species exhibit enlarged fermentative compartments, such as the rumen or caecum, which support high microbial density and enhanced fiber fermentation. In contrast, carnivores display shorter intestinal tracts with reduced fermentative capacity. Despite interspecies variation, dominant bacterial phyla are conserved, suggesting functional redundancy across mammalian hosts while maintaining ecological specialization [62].

The gut microbiota contributes to host physiology through an extensive range of metabolic, structural, and immunological functions. A major metabolic role involves the fermentation of undigested polysaccharides and resistant starches to SCFAs [2]. Butyrate serves as the principal energy source for colonocytes, enhances tight-junction integrity, and exerts anti-inflammatory effects by inhibiting HDACs. Acetate and propionate, on the other hand, influence hepatic gluconeogenesis and lipid metabolism, linking microbial fermentation to systemic energy balance [6].

In addition to carbohydrate metabolism, gut microbes synthesize vitamins B and K, can transform bile acids into secondary derivatives with signaling roles, and regulate tryptophan metabolism and serotonin production [26]. These processes contribute to immune and neuroendocrine regulation, exemplifying the microbiota’s integration into the gut–brain and gut–immune axes.

From an immunological perspective, commensal microbiota shapes both innate and adaptive immunity. They promote the maturation of intestinal lymphoid structures, enhance IgA secretion, and regulate T helper cell differentiation. Recognition of microbial molecular patterns by PRRs, such as Toll-like receptors (TLRs), NOD-like receptors (NLRs), RIG-I-like receptors (RLRs), Aim2-like receptors (ALRs), and C-type lectin-like receptors (CLRs) [63], enables controlled immune activation while maintaining tolerance to non-pathogenic taxa [64]. Experiments in germ-free animals consistently demonstrate that microbial absence leads to underdeveloped Peyer’s patches, reduced lamina propria lymphocytes, and diminished antibody diversification [65].

The composition of the gut microbiota is shaped by both intrinsic host factors and external environmental influences. Early colonization depends on the mode of birth, exposure to maternal microbiota, and diet—particularly the presence of milk oligosaccharides that selectively promote *Bifidobacteria* [26]. In adulthood, diet remains the most potent modulator: fiber-rich, plant-based diets support saccharolytic species such as *Prevotella* and *Ruminococcus*, while high-fat, high-protein diets increase bile-tolerant and proteolytic taxa, including *Bilophila wadsworthia* [7].

Antibiotics, xenobiotics, and stress perturb microbial homeostasis, often reducing diversity and enabling opportunistic overgrowth [58]. Host genetics also influence microbial composition through variations in mucin glycosylation, immune signaling, and epithelial antimicrobial peptides. Physiological factors such as age and circadian rhythm add further complexity; aging is typically associated with reduced microbial diversity and loss of SCFA-producing taxa, whereas environmental exposure—“microbial rewilding”—has been shown to restore regulatory immune networks in animal models [57].

Beyond diet and chemicals, gut motility itself is a major ecological filter that shapes microbial assembly, metabolism, and spatial niches along the intestine. Whole-gut and segmental transit times strongly correlate with community composition and activity and can confound “diet–microbiome” associations if not accounted for [66]. Peristaltic flow structures habitats and enforces competitive exclusion; when motility is reduced, slower washout permits persistence and overgrowth of taxa adapted to low-flow environments, whereas normal contractile activity promotes dynamic turnover and resilience [67]. Mechanistically, microbial signals—including SCFAs, secondary bile acids, and tryptophan catabolites—act on epithelial, enteroendocrine, immune, and enteric nervous system targets (e.g., GPCRs, TLRs, AhR) to modulate secretion, neuromuscular excitability, and serotonergic tone, thereby closing a bidirectional loop in which microbial metabolites regulate gastrointestinal motility, while transit dynamics reciprocally influence microbial colonization and ecosystem composition [68,69]. Clinically, dysmotility states illustrate these couplings: fecal transplants from patients with slow-transit constipation transfer delayed transit and reduced SCFAs/secondary bile acids to recipient mice, implicating metabolite deficits in hypomotility [70]. Conversely, taxa producing long-chain (and other) fatty acids have been linked to accelerated colonic transit in stress-susceptible models, indicating metabolite–motor specificity [71]. In humans, constipation and diarrhea phenotypes show partially reproducible microbial signatures, but heterogeneity remains high, underlining the need for standardized motility phenotyping alongside multi-omics [10]. As a practical implication, interventions that normalize transit—from targeted probiotics/prebiotics that enhance SCFA output to lifestyle measures such as physical exercise—may realign community structure and inflammatory tone simultaneously [69,72].

Although the core functional capacities of the gut microbiome are conserved across mammals, taxonomic composition varies according to diet, gut morphology, and physiology. Rodents, the most frequently used laboratory models, exhibit a dominance of *Lactobacillus*, *Muribaculaceae*, and *Clostridium* clusters IV and XIVa, reflecting their cecal fermentation strategy [57,58]. Pigs display a microbiota composition and fermentation profile most similar to humans, with comparable *Firmicutes*/*Bacteroidetes* ratios and abundant *Prevotella*, *Ruminococcus*, and *Faecalibacterium* species [59]. Consequently, gnotobiotic pig models are highly relevant for translational microbiome–immune research.

Ruminants, including cattle and sheep, rely on foregut fermentation, with *Ruminococcus*, *Fibrobacter*, and methanogenic archaea (*Methanobrevibacter* spp.) dominating ruminal microbiota [50]. Despite the anatomical specialization, the production of SCFAs and regulation of host immunity follow similar principles to those observed in monogastric species. Carnivores such as dogs and cats exhibit a distinct microbial pattern characterized by reduced diversity and enrichment of proteolytic taxa (*Clostridium perfringens*, *Peptostreptococcus*, *Fusobacterium*) adapted to protein-rich, low-fiber diets [61].

**Figure 4:**
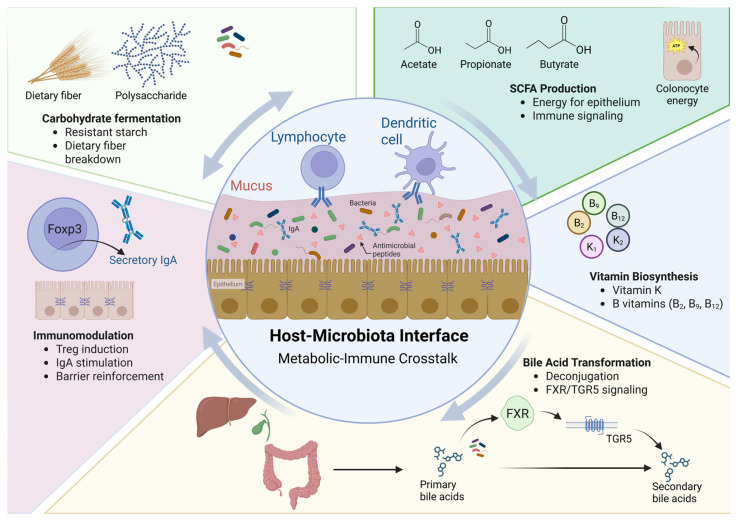
Functional roles of the gut microbiota in mammalian hosts (Created with BioRender.com) Diagram illustrating key microbial functions: fermentation of complex polysaccharides, production of SCFAs, vitamin synthesis, bile acid transformation, and immunomodulation (Treg induction, IgA stimulation, epithelial barrier reinforcement). Central arrows depict bidirectional host–microbiota interactions linking metabolism and immunity.

In summary, while microbial composition varies among mammals, functional convergence—particularly in carbohydrate fermentation, vitamin synthesis, and immune regulation—demonstrates the evolutionary stability of the host–microbe symbiosis (Figure 4). Comparative analyses across species not only elucidate fundamental mechanisms of coadaptation but also enhance the translational relevance of animal models for human and veterinary health research [8,59].

Table 2 summarizes the major compositional and functional characteristics of the gut microbiome across representative mammalian species.

## 5. Host Immune System Overview

Metazoans, defined as multicellular animal organisms, perform essential biological functions—nutrition, relation, and reproduction—that inevitably involve continuous interaction with microorganisms. These host–microbe relationships are fundamental determinants of the host physiology, immune reaction, and overall fitness. The nature of these interactions spans a continuum from highly antagonistic to mutually beneficial and is determined by the evolutionary adaptations of both the host and the microbial species. Depending on the outcome of the interaction, microorganisms may function as pathogens, reducing the host fitness, or as commensals, exerting neutral or beneficial effects on the host. It is now well established that the host immune system plays a central role in managing these relationships—protecting against pathogens while maintaining tolerance to commensals, as inappropriate immune activation against symbiotic microbiota can trigger chronic inflammation and tissue damage [75]. Understanding these interactions is particularly relevant in the GIT, where the gut microbiota exerts profound influences on host nutrition, immune regulation, and overall health.

### 5.1. Innate Defenses of the Intestinal Mucosa

The intestinal mucosa represents the largest immunological interface between the host and the external environment. It relies on multiple layers of defense that collectively sustain the adequate functioning of the immune system. The first line of protection is provided by the epithelial barrier, consisting of polarized epithelial cells interconnected by tight junctions. These cells not only restrict the translocation of luminal antigens but also actively participate in immune sensing through PRRs [76,77]. Controlled activation of PRRs enables the recognition of pathogen-associated molecular patterns (PAMPs) and damage-associated molecular patterns (DAMPs) while preventing excessive inflammation.

Secretory mucins produced by goblet cells form a structured mucus layer that limits direct contact between microbes and the epithelial surface [78]. The outer mucus layer serves as a habitat for commensals, while the inner layer remains sterile. Embedded within the epithelial lining, Paneth cells release antimicrobial peptides (AMPs) such as α-defensins, lysozyme, and RegIIIγ, which shape microbial populations and prevent bacterial encroachment [79]. These AMPs act synergistically with immunoglobulin A (IgA) and secretory phospholipase A2 to maintain the spatial segregation of microbiota and host tissues.

Innate immune cells located beneath the epithelium—dendritic cells (DCs), macrophages, and innate lymphoid cells (ILCs)—constitute the second layer of mucosal defense. DCs sample luminal antigens through trans-epithelial dendrites and migrate to mesenteric lymph nodes to initiate adaptive responses [80,81]. Macrophages maintain local tolerance by clearing apoptotic cells and secreting anti-inflammatory cytokines such as IL-10 [82]. Type 3 ILCs, characterized by RORγt expression, produce IL-22, which enhances epithelial repair and AMP secretion [83]. Together, these components establish a dynamic equilibrium between immune activation and tolerance.

### 5.2. Adaptive Immune Compartment and GALT Organization

The GALT represents the organized adaptive arm of the mucosal immune system. It includes Peyer’s patches, isolated lymphoid follicles, mesenteric lymph nodes, and diffuse lymphoid cells within the lamina propria [64]. Peyer’s patches, located primarily in the distal ileum, function as inductive sites for mucosal immunity and oral tolerance. They are covered by the follicle-associated epithelium (FAE), which contains microfold (M) cells specialized in transcytosis of luminal antigens and microbes to the underlying antigen-presenting cell. This process enables antigen sampling without compromising epithelial integrity.

Within Peyer’s patches, naive B cells undergo somatic hypermutation and class-switch recombination, leading to the generation of IgA-secreting plasma cells. Secretory IgA (sIgA) is transported across the epithelium via the polymeric immunoglobulin receptor (pIgR) and plays a crucial role in immune exclusion—coating microbial surfaces and preventing pathogen adherence [84]. Studies comparing germ-free and conventionalized animals have shown that microbial exposure drives IgA diversification and expansion of plasma cells in the lamina propria [85,86]. IgA responses are thus tightly linked to commensal colonization and dietary antigen exposure.

T cell subsets in the intestine further ensure immune balance. Conventional CD4^+^ T cells (Th1, Th2, Th17, and Treg) populate the lamina propria, while intraepithelial lymphocytes (IELs)—CD8^+^ αβ and γδ T cells—reside between epithelial cells and provide immediate effector functions. Microbial colonization profoundly influences IEL repertoire and activation [87], while chemokines such as CCL25 coordinate their recruitment and maintenance [88]. Commensal bacteria, including segmented filamentous bacteria (SFB) in mice, promote Th17 differentiation and IL-17 production. Th17 cells contribute to mucosal defense by enhancing neutrophil recruitment and epithelial barrier protection but may also drive chronic inflammation when dysregulated.

In parallel, MAIT cells form a conserved lineage that recognizes riboflavin-derived microbial metabolites presented by MR1 [89], and they play critical roles in mucosal repair, infection control, and metabolic regulation, and recent studies indicate that their abundance and activity are closely tied to microbiota composition and integrity [90].

Mucosal immune responses are not restricted to a single anatomical site but are integrated through the common mucosal immune system. Lymphocytes activated within GALT can recirculate through the bloodstream and home to distant mucosal tissues, including the respiratory and urogenital tracts, through the coordinated expression of adhesion molecules and chemokine receptors. This coordinated trafficking provides a mechanistic basis for communication among gastrointestinal, respiratory, and urogenital mucosae, thereby supporting immune balance across multiple mucosal surfaces (Table 3).

### 5.3. Immune Tolerance and Regulation

A defining feature of the intestinal immune system is its ability to distinguish between harmless antigens and harmful pathogens. This tolerance–defense balance is maintained through multiple regulatory mechanisms. Tregs secrete IL-10 and TGF-β, which suppress excessive inflammatory signaling, while tolerogenic DCs drive the induction of antigen-specific Tregs in the presence of retinoic acid and microbial metabolites [75,80,82]. Commensal bacteria such as *Bacteroides fragilis* contribute to this process through polysaccharide A (PSA), which promotes IL-10 production and prevents colitis in murine models [85].

Moreover, epithelial and immune cells engage in a continuous crosstalk that reinforces barrier integrity. Cytokines like IL-22 and IL-18 promote epithelial regeneration and AMP production, while IL-1β and TNF-α coordinate local inflammation and repair [105,106,107]. Dysregulation of these pathways—whether by loss of microbial diversity, genetic defects in PRR signaling, or impaired IgA secretion—can precipitate inflammatory bowel diseases (IBD), allergies, and autoimmune disorders.

Microbial metabolites further reinforce tolerance. SCFAs generated by bacterial fermentation of dietary fibers, enhance Treg development through HDAC inhibition and G-protein-coupled receptor signaling [108]. In addition, microbial and host metabolites can epigenetically modulate immune gene expression, illustrating the fine-tuned integration of metabolic and immune regulation [109]. This multilayered regulation ensures that protective immunity against pathogens coexists with tolerance to commensals and dietary antigens.

### 5.4. Comparative and Translational Perspectives

Although the fundamental architecture of the immune system is conserved across mammals, species-specific variations in gut morphology and microbial exposure influence immune outcomes. Mammalian and avian models show how different GALT structures act as primary lymphoid sites for B cell diversification [84]. In mice, the compact intestinal length and presence of cecal Peyer’s patches make them ideal for mechanistic studies of mucosal immunity, particularly under germ-free or gnotobiotic conditions [58]. Pigs, by contrast, possess multifollicular lymphoid clusters throughout the small and large intestines, closely mirroring human GALT organization and providing high translational relevance [59]. Comparative immunology thus provides essential context for interpreting microbiota–immune interactions across species.

Advanced gnotobiotic and in vitro systems now permit controlled dissection of host–microbe interactions while reducing animal use [110]. Multi-omics integration and synthetic microbiomes allow identification of conserved pathways governing mucosal immunity and tolerance [111].

Notably, recent work emphasizes the involvement of unconventional immune populations (ILCs, MAIT cells, γδ T cells) in coordinating epithelial barrier repair and systemic immune tone [83]. These mechanisms underscore the intimate coevolution of the immune system and microbiota, forming a functional meta-organism essential for host health.

## 6. Interaction Between the Gut Microbiome and Host Immunity

The gut microbiome and the host immune system engage in a continuous, bidirectional dialogue that determines intestinal and systemic immune balance. Far from being passive occupants, commensal microorganisms actively instruct immune development, modulate effectors and regulatory pathways, and participate in tissue repair. Conversely, the host immune system shapes the microbial landscape through secretory antibodies, antimicrobial peptides, and cytokine-mediated control mechanisms [76,105,112,113]. The outcome of this dynamic interaction is a finely tuned equilibrium that protects against infection while maintaining tolerance to the vast community of symbionts [114,115,116].

### 6.1. Molecular Dialogue Between Microbes and Host Cells

Communication begins at the epithelial interface through PRRs, which detect conserved microbial structures. The TLRs and NLRs expressed on epithelial cells, DCs, and macrophages recognize molecules such as lipopolysaccharide, flagellin, and peptidoglycan [117]. Controlled PRR signaling induces low-level NF-κB activation, resulting in tonic expression of cytokines (IL-1β, IL-6, TNF-α) and antimicrobial peptides that preserve epithelial turnover and defense without provoking overt inflammation [76].

Commensal sensing is spatially regulated: TLR5 on the basolateral membrane detects flagellin only when bacteria breach the mucus barrier, while apical TLR engagement by luminal ligands can trigger tolerance via inhibition of MyD88 signaling [118]. Similarly, NOD2, the cytosolic receptor mutated in Crohn’s disease, recognizes muramyl dipeptide and promotes Paneth-cell defensin secretion [119]. These mechanisms demonstrate that innate immune receptors operate not as simple alarms but as contextual sensors that interpret microbial signals to maintain mucosal balance.

While direct cell-to-cell contact and soluble factors are well-established mechanisms of host–microbe interaction, extracellular vesicles (EVs) have emerged as a critical third mode of communication [120]. According to the Minimal Information for Studies of Extracellular Vesicles (MISEV2023) guidelines, EVs are defined as particles naturally released from cells that are delimited by a lipid bilayer and cannot replicate [121]. In the context of the gut microbiome, it is essential to distinguish between mammalian EVs and bacterial EVs (BEVs). BEVs are produced by both Gram-negative bacteria (often termed outer membrane vesicles or OMVs, sized 20–250 nm) and Gram-positive bacteria (membrane vesicles or MVs, sized 20–400 nm) [122,123,124,125]. Unlike mammalian exosomes, which originate from the endosomal pathway, or ectosomes, which bud from the plasma membrane [122], BEVs are generated through membrane blebbing or explosive cell lysis, encapsulating periplasmic and cytoplasmic components [122,126,127].

EVs serve as stable transport vehicles that protect their cargo—including proteins, lipids, nucleic acids (DNA, mRNA, miRNA, and 16S rRNA), and metabolites—from enzymatic degradation in the extracellular environment [128,129]. This stability allows microbiota-derived EVs to penetrate the sterile mucus layer that typically segregates bacteria from host tissue, enabling them to interact directly with intestinal epithelial cells (IECs) and resident immune cells. Furthermore, these vesicles can enter the systemic circulation and reach distant organs, thereby influencing the gut–brain axis, metabolic regulation, and pulmonary immunity [122,130].

BEVs play a dual role in immune modulation, acting as either pro-inflammatory or anti-inflammatory agents depending on the parent bacterial strain. EVs derived from the mucin-degrading bacterium *Akkermansia muciniphila* have been shown to enter IECs and upregulate the expression of tight junction proteins such as zonula occludens-1 (ZO-1) and occludin, thereby reducing gut permeability and ameliorating colitis symptoms [122,131,132]. Similarly, EVs from the probiotic *Escherichia coli* Nissle 1917 can activate NOD1 signaling to enhance epithelial barrier function and induce the expression of anti-inflammatory cytokines like IL-10 [133,134].

Commensal BEVs, such as those from *Bacteroides fragilis*, contain PSA, which interacts with DCs to promote the differentiation of regulatory T cells (Tregs) and suppress pro-inflammatory T helper 17 (Th17) responses [123,135]. Conversely, recent evidence suggests that gut microbiota-derived small EVs can “train” neutrophils, enhancing their pro-inflammatory capacity via the TLR4/MyD88 signaling pathway, a mechanism that may contribute to exaggerated immune responses in sepsis [136,137].

Disruptions in the profile and cargo of gut-derived EVs are associated with various pathologies:

- Inflammatory Bowel Disease: The proteomic profile of intestinal EVs changes significantly in pediatric IBD, showing correlations with disease severity and specific microbial taxa like *Faecalibacterium prausnitzii* [138]. BEVs in IBD can carry pro-inflammatory factors that exacerbate mucosal damage [128].
- Metabolic Disorders: BEVs have been implicated in the pathogenesis of type 2 diabetes. For instance, EVs from *Pseudomonas panacis* can block insulin signaling in skeletal muscle and adipose tissue, promoting glucose intolerance [120]. Conversely, *A. muciniphila* EVs have shown potential in alleviating obesity-induced metabolic dysfunction [131].
- Cancer: BEVs can influence the tumor microenvironment. In colorectal cancer, specific bacterial EVs may promote cell proliferation or, conversely, be engineered to deliver therapeutic agents due to their ability to target specific tissues [122,137].

**Figure 5:**
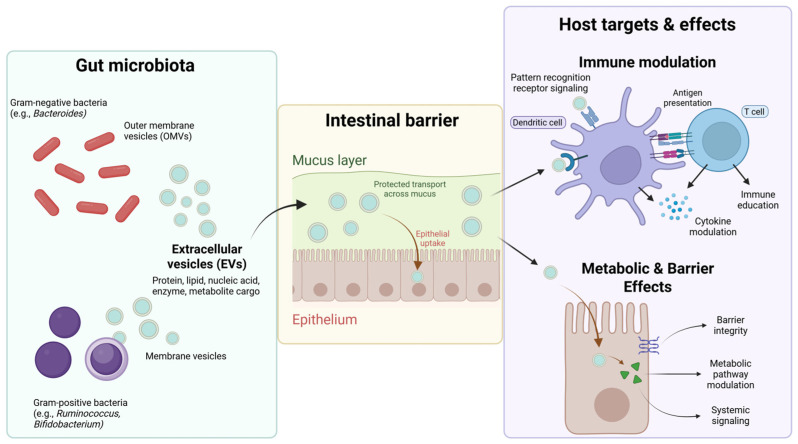
Microbiota-derived extracellular vesicles as mediators of host–microbe communication (Created with BioRender.com). The schematic illustrates the release of EVs by gut microorganisms and their interactions with epithelial and immune cells through the transfer of microbial antigens, nucleic acids, metabolites, and signaling molecules involved in barrier regulation, immune modulation, and intercellular communication.

As depicted in Figure 5, bacterial extracellular vesicles function as nanoscale vectors that bridge spatial separation between luminal microbes and host tissues. By traversing the mucus layer and interacting with epithelial and immune cells, BEVs integrate microbial sensing, antigen presentation, cytokine modulation, and barrier regulation within a unified host–microbiota communication axis.

The study of microbiome-derived EVs offers new avenues for “postbiotic” therapies, where EVs are used to deliver beneficial bacterial effects without the risks associated with administering live microorganisms, particularly in immunocompromised hosts [122,139]. However, rigorous standardization of isolation methods (e.g., size exclusion chromatography, ultracentrifugation) and characterization is required to distinguish BEVs from non-vesicular extracellular particles (NVEPs) and host-derived vesicles, as emphasized by the MISEV2023 guidelines [106].

### 6.2. Microbiota-Derived Metabolites as Regulators of Host Immunometabolism

#### 6.2.1. Bile Acid Metabolism and Host Interaction

Primary bile acids (BAs), cholic acid (CA) and chenodeoxycholic acid (CDCA), are synthesized from cholesterol in the liver, conjugated with glycine or taurine, and secreted into the intestine. While most conjugated BAs are reabsorbed via enterohepatic circulation, approximately 5% reach the colon, where they undergo extensive microbial biotransformation [140,141,142,143].

The most prominent microbial pathway is 7α-dehydroxylation, converting primary BAs into secondary BAs such as deoxycholic acid (DCA) and lithocholic acid (LCA). This multistep anaerobic process is encoded by the *bai* operon and is restricted to a limited subset of *Firmicutes*, including *Clostridium scindens* and *C. hylemonae* [141,143,144,145]. Reconstruction of this pathway identified a minimal enzymatic set (BaiB, BaiCD, BaiA2, BaiE, BaiF, BaiH) sufficient for CA-to-DCA conversion, employing a unique redox mechanism involving transient oxidation of the steroid A/B rings to facilitate reductive elimination of the 7α-hydroxyl group [143,146].

In addition to dehydroxylation, gut bacteria modify BA pools through bile salt hydrolase (BSH)-mediated deconjugation and position-specific oxidation or epimerization reactions, substantially increasing BA chemical diversity and receptor specificity [140,142,144]. Microbiota-derived secondary BAs act as potent signaling molecules via host BA receptors, particularly the nuclear farnesoid X receptor (FXR) and the membrane G-protein-coupled receptor TGR5 (GPBAR1) [140,141,144]. Specific BA metabolites directly regulate adaptive immunity: 3-oxo-LCA suppresses Th17 cell differentiation, whereas isoallo-LCA promotes regulatory T cell (Treg) induction through mitochondria-dependent transcriptional programs [140]. These findings establish BA metabolism as a direct microbial control axis over the appropriate functioning of the host’s immune system. BA signaling also integrates metabolic and cardiovascular regulation. TGR5 activation stimulates glucagon-like peptide-1 (GLP-1) secretion, improving glucose tolerance and insulin sensitivity [141,147]. Disruption of microbiota–BA crosstalk has been implicated in type 2 diabetes and diabetic kidney disease (DKD), where altered microbial communities shift BA profiles toward pro-inflammatory states; restoration of BA receptor signaling has shown renoprotective effects in experimental models [147]. Clinically, elevated circulating DCA levels have been associated with increased cardiovascular risk in individuals with newly diagnosed type 2 diabetes [148].

#### 6.2.2. Tryptophan Metabolism and Indole Derivatives

Tryptophan (Trp) is an essential amino acid extensively metabolized by the gut microbiota into indole and indole-derived compounds, including indole-3-acetic acid (IAA), indole-3-lactic acid (ILA), indole-3-propionic acid (IPA), and oxindole derivatives [149,150,151].

A central mechanism underlying indole bioactivity is activation of the aryl hydrocarbon receptor (AhR). Multiple microbiota-derived indoles function as endogenous AhR ligands at physiologically relevant concentrations, supporting epithelial barrier integrity, IL-22-mediated antimicrobial responses, and mucosal immune balance [150,152,153].

Several indoles exert pronounced anti-inflammatory effects. ILA, predominantly produced by *Lactobacillus* species, attenuates intestinal inflammation and enhances epithelial resilience [149,154]. Importantly, ILA promotes microbial cross-feeding, increasing the abundance of secondary indole-producing taxa such as *Clostridium*, thereby amplifying production of IPA and IAA with cumulative protective effects in colitis models [154].

Indole signaling also modulates systemic inflammatory conditions. In graft-versus-host disease (GvHD), microbiota-derived indoles preserve epithelial integrity and limit immune-mediated tissue injury through type I interferon-dependent pathways [142]. However, Trp metabolism is context-dependent: indole absorbed systemically is converted in the liver to indoxyl sulfate, a uremic toxin implicated in nephrotoxicity and cardiovascular disease, particularly in chronic kidney disease [142].

#### 6.2.3. Short-Chain Fatty Acids

SCFAs—acetate, propionate, and butyrate—are produced through microbial fermentation of dietary fibers. Their synthesis involves distinct metabolic routes, including the Wood–Ljungdahl pathway (acetate), succinate and acrylate pathways (propionate), and acetyl-CoA condensation (butyrate) [145,155,156,157].

SCFAs signal primarily through the G-protein-coupled receptors FFAR2 (GPR43) and FFAR3 (GPR41), regulating inflammation, enteroendocrine hormone secretion (GLP-1, PYY), and systemic energy metabolism [24,158,159,160]. At the immune level, SCFAs promote regulatory T cell differentiation, modulate macrophage polarization, and suppress pro-inflammatory cytokine production via receptor-dependent signaling and histone deacetylase inhibition (HDAC) [161].

Clinically, SCFAs are associated with improved insulin sensitivity and reduced chronic inflammation, suggesting protective roles in metabolic syndrome and diabetic nephropathy [147,161]. However, human intervention studies demonstrate high inter-individual variability; systematic reviews indicate that dietary fiber supplementation does not uniformly increase fecal SCFAs concentrations, likely reflecting differences in microbiota composition and fiber fermentability [147,162].

#### 6.2.4. Microbiota–Immune Chemical Crosstalk and Adaptive Immune Regulation

Gut microbial communities engage in extensive chemical cross-feeding, in which the metabolites produced by one taxon serve as substrates or regulatory signals for others, shaping community-level metabolic outputs and ecosystem stability [153,154]. Multispecies studies demonstrate that specialized secondary metabolites can actively modulate neighboring microbial metabolism, reinforcing functional resilience within the microbial network [142].

Collectively, the gut microbiota functions as a metabolically active endocrine system. Through transformation of dietary substrates and host-derived molecules, microbial metabolites exert systemic control over immune differentiation, metabolic regulation, and disease susceptibility [140,141,153].

The chemical signaling directly programs adaptive immunity. The development and maturation of gut lymphoid structures, including Peyer’s patches and isolated lymphoid follicles, require microbial colonization [146]. Germ-free animals exhibit reduced follicle size, decreased germinal-center activity, and markedly lower levels of secretory IgA [163]. Individual commensal taxa have specialized immunomodulatory roles: *Bacteroides fragilis* promotes Treg expansion via PSA-dependent IL-10 production [85,164], whereas segmented filamentous bacteria (SFB) induce Th17 differentiation and strengthen epithelial defense [165,166].

At the epithelial surface, IELs provide immediate cytotoxic responses and help preserve epithelial integrity. Their abundance and activation state are influenced by microbial colonization, with germ-free mice showing decreased CD8α^+^ αβ T-cell frequencies [167,168,169]. MAIT cells, which recognize vitamin B2 metabolites presented by MR1 molecules, are nearly absent in germ-free conditions and reconstitute only after colonization with riboflavin-synthesizing bacteria [83,90,170,171].

Secretory IgA acts as the dominant antibody at mucosal surfaces, coating commensals to prevent epithelial translocation while facilitating immune inclusion rather than exclusion. Continuous antigen sampling drives polyclonal IgA responses that limit bacterial overgrowth yet preserve diversity [172]. Thus, adaptive immune education by the microbiota ensures tolerance to symbionts and readiness against pathogens.

Collectively, microbiota-derived metabolites form interconnected signaling networks that regulate host immune and metabolic homeostasis. The major metabolite classes, their microbial origins, host targets, and immunological effects are summarized in Table 4.

### 6.3. Dysbiosis, Immune Dysregulation and Systemic Disease

Evidence linking gut dysbiosis to systemic disease derives from multiple experimental and clinical research approaches. Mechanistic insights originate primarily from in vitro studies, germ-free and gnotobiotic animal models, whereas human evidence is largely based on observational cohorts and a limited number of intervention trials. Consequently, the strength of causal inference varies across disease contexts and should be interpreted according to the underlying level of evidence.

When microbial diversity declines or beneficial taxa are lost, this homeostatic dialogue collapses. Dysbiosis can arise from antibiotics, Western-style diets, or host genetic susceptibility [115]. A reduction in beneficial taxa can alter the production of key microbial metabolites, including short-chain fatty acids and BA derivatives, which are essential for epithelial integrity and immune tolerance. Consequently, impaired Treg differentiation, compromised barrier function, and enhanced pro-inflammatory signaling promote chronic mucosal inflammation [174].

In inflammatory bowel disease (IBD), dysbiosis is characterized by expansion of facultative anaerobes such as *Enterobacteriaceae* and depletion of anti-inflammatory commensals, including *Faecalibacterium prausnitzii*, correlating with increased Th1 and Th17 immune activity [175,176,177,178,179,180,181,182,183,184]. Similarly, low microbial richness in early life has been linked to increased risk of asthma and atopy due to insufficient induction of tolerogenic pathways [185,186].

Beyond the intestine, microbial signals influence systemic immunity. Translocation of bacterial products such as LPS during dysbiosis can trigger low-grade endotoxemia, contributing to obesity-related inflammation and metabolic syndrome [173,187]. Experimental animal studies and translational human investigations suggest that dysbiosis may increase caloric extraction from otherwise indigestible substrates, alter bile-acid pools and SCFA profiles, and promote endotoxemia and low-grade inflammation that impair insulin sensitivity and adipose-tissue homeostasis [188,189,190,191]. Large-scale metagenomic work further shows that obesity is characterized not only by taxonomic shifts but also by rewired microbial co-abundance networks and pathways, underscoring that interaction structure—not just who is present—matters for metabolic phenotype [192]. While some cohorts report higher *Firmicutes* and lower *Bacteroidetes* in obesity, a recent systematic review highlights substantial heterogeneity across studies, cautioning against a single “obese microbiome” signature [193]. Rather than a uniform taxonomic pattern, dietary context and metabolic output determine inflammatory tone and metabolic resilience. Observational and interventions studies indicate that diet quality and the source of fats and proteins influence microbial ecosystem structure—affecting SCFA output, bile-acid transformation, and inflammatory tone—and thereby shape obesity risk [194,195]. Causality in humans remains difficult to prove, but converging evidence supports targeted microbiome modulation (prebiotics, probiotics/synbiotics, diet personalization) as a component of 3P (predictive, preventive, personalized) strategies for metabolic health [196,197].

Microbiota-derived tryptophan and bile-acid metabolites also affect the blood–brain barrier and microglial activation, suggesting a far-reaching gut–immune–brain axis [198]. These findings underscore that immune imbalance originating in the gut can propagate systemically, linking local dysbiosis to multi-organ diseases. Emerging evidence highlights the gut–brain–immune axis as a multidirectional communication network linking microbial metabolites, vagal and endocrine signaling, and neuroimmune circuits. Observational human studies together with experimental animal models support the concept of a gut–brain–immune axis linking dysbiosis with altered vagal signaling, HPA-axis dynamics, cytokine milieus, and symptom burden [199,200,201,202,203,204,205]. Preclinical studies and early clinical investigations suggest that targeting age-related dysbiosis through probiotics, dietary fiber, or microbial metabolites may therefore support neuroimmune resilience and promote healthy aging [206]; however, robust long-term clinical evidence remains limited. Building on this framework, convergent evidence indicates that gut–brain–immune communication operates through multiple, partially overlapping routes—microbial metabolites (e.g., indoles/tryptophan catabolites, secondary BAs), neuroactive molecules, and neural and endocrine relays—that together calibrate central inflammation, neurotransmission, and behavior [207,208,209,210]. Microbial regulation of serotonergic tone via host tryptophan metabolism is a key node: shifts in microbial enzymes and host kynurenine pathway activity reshape serotonin availability and microglial reactivity, with downstream effects on mood, cognition, and pain processing [211,212]. Historical and ethological perspectives further underscore that host–microbe co-adaptation and microbial transfer across the lifespan shape sociality and stress responsivity, reinforcing the concept of a “microbiota–gut–vagus–brain” continuum [213]. From a therapeutic standpoint, psychobiotics and microbiota-targeted strategies (dietary fiber/prebiotics, defined probiotics/synbiotics, and postbiotic metabolites) are being investigated as adjunctive approaches and may complement standard care, although clinical evidence remains limited and heterogeneous [212,214]. Integrative 3P medicine approaches—combining mechanistic biomarkers, longitudinal monitoring, and personalized microbiome modulation—are therefore poised to mitigate inflammaging-related neuroimmune drift and support healthy cognitive and emotional aging [209,212].

### 6.4. Therapeutic and Translational Perspectives

Restoring microbial balance has emerged as a therapeutic goal in immunological disorders. Disruption of microbial diversity alters SCFA and BA signaling, impairs epithelial barrier integrity, and amplifies inflammatory cascades. These alterations reinforce immune dysregulation and perpetuate tissue injury. Accordingly, therapeutic strategies increasingly aim to re-establish ecological stability and immunometabolic equilibrium.

Microbiota-directed interventions range from selective microbial supplementation to whole-ecosystem restoration, with their mechanisms, immunological effects, and translational applications summarized in Table 5.

Despite the therapeutic potential of microbiota-based interventions, successful colonization of exogenously administered microorganisms remains a major challenge. The resident microbiota exhibits substantial colonization resistance through competition for nutrients, occupation of ecological niches, production of antimicrobial compounds, and host-mediated immune mechanisms. Consequently, many probiotic strains demonstrate only transient persistence within the gastrointestinal tract, and therapeutic efficacy may vary considerably among individuals. These factors contribute to the heterogeneous outcomes observed across clinical studies and highlight the need for personalized microbiome-targeted approaches.

In parallel to microbiota-centered therapies, biological immunomodulators such as anti-TNF, anti-IL-12/23, or JAK inhibitors remain cornerstones in the treatment of immune-mediated diseases. Recent findings indicate that these biologics also reshape the gut microbiota, being associated with shifts toward a more diverse microbial profile in responders [115].

Oral IgY preparations represent an emerging microbiota-compatible adjunct. By providing pathogen-specific neutralization without disrupting commensal communities, IgY therapy offers targeted mucosal protection with a proposed lower risk of selecting resistance, although clinical data remain limited [229].

Advances in in vitro and in silico platforms—including dynamic gut simulators, organoid co-cultures, and systems-biology modeling—enable controlled dissection of host–microbe interactions and accelerate biomarker discovery while aligning with the 3Rs principle [73,110,111,230,231]. These models enable reproducible evaluation of host–microbiota interactions and preclinical screening of dietary, probiotic, and immunomodulatory interventions under physiologically relevant conditions.

The cumulative evidence affirms that the gut microbiome is not a passive commensal community but an active immunological organ that continually tunes host defenses and tolerance. Its perturbation underlies a continuum of immune-mediated pathologies, making the restoration of microbial homeostasis a central goal in precision medicine.

## 7. Current Research and Findings

Recent years have seen a dramatic expansion of experimental and translational work exploring the gut microbiota–immune interface. Metagenomic surveys and microbial–host correlation studies have mapped how individual taxa, microbial networks, and metabolites influence immune regulation and responses across life stages [73,74]. These efforts have shifted the field from descriptive ecology to mechanistic insight, revealing that commensal communities function as metabolic and immunological organs whose composition determines the amplitude and tone of immune responses [232,233].

Large-scale cohort analyses and twin studies have highlighted the joint contribution of genetic and environmental factors in shaping microbial and immune diversity. Heritable microbial modules linked to mucosal immune genes suggest a co-evolutionary relationship between host genotype and microbial metabolism [234,235]. Diet, antibiotics, and xenobiotics could rapidly remodel these networks, providing mechanistic explanations for geographic and lifestyle-associated differences in immune reactivity [236,237].

Functional profiling has uncovered how the gut microbiota modulates innate and adaptive immunity through metabolite-mediated pathways. SCFAs and tryptophan-derived indoles act as epigenetic regulators of cytokine expression, whereas microbial modification of BAs influences macrophage polarization and IgA synthesis [153,174]. Multi-omics integration now links these metabolites to systemic outcomes, including metabolic and cardiovascular inflammation [238,239].

Parallel studies focus on microbiota-directed therapeutics. Engineered bacterial consortia and next-generation probiotics are being designed to deliver anti-inflammatory metabolites or to compete with pathobionts in vivo [240,241]. Preclinical and early clinical data indicate that targeted microbiome restoration may enhance the efficacy of immunotherapies and vaccines by improving antigen presentation and mucosal antibody production [242,243]. Beyond bacterial members, attention has turned to the mycobiome [244,245,246] and virome [247,248,249], whose interactions with mucosal immunity are increasingly recognized as modulators of disease susceptibility [250,251,252].

Despite these advances, reproducibility and ethical considerations remain major challenges. Systems-biology approaches combining in vitro gut simulators, organoid co-cultures, and computational modeling provide controlled environments to study microbe–immune dynamics while reducing animal use [111,206]. Collectively, these developments demonstrate a maturing field that integrates ecological, immunological, and translational perspectives to delineate causal mechanisms underlying host–microbiota symbiosis.

ClinicalTrials.gov contains a rapidly expanding portfolio of studies investigating FMT, probiotics, postbiotics, and other microbiota-directed interventions. This growing clinical pipeline reflects the increasing translation of microbiome science into preventive, immunological, and therapeutic applications.

## 8. Conclusions and Future Directions

Despite the impressive progress achieved in understanding the gut microbiota–immune axis, several challenges continue to limit translational application. The main obstacle remains the high inter-individual variability in microbiota composition, which complicates the reproducibility of intervention outcomes and the identification of universal biomarkers of immune modulation [73,74]. To address this, future research should adopt standardized analytical pipelines and longitudinal multi-omics profiling to capture dynamic microbial and immunological responses at the individual level.

Several methodological and biological limitations must also be considered when interpreting microbiome–immune associations. Most microbiome studies rely on relative abundance data, making analyses susceptible to compositionality effects that can obscure true biological changes [253]. In addition, differences in sampling procedures, DNA extraction methods, sequencing platforms, and bioinformatic pipelines may introduce batch effects that complicate comparisons across studies [254,255]. Microbiome composition is further influenced by numerous confounding variables, including diet, medication use (particularly antibiotics, proton-pump inhibitors, and immunomodulatory drugs), age, lifestyle, and geographic location, which may contribute substantially to the heterogeneity observed between cohorts [1,2,234,235,236]. Importantly, many human studies remain observational, limiting causal inference and raising the possibility of reverse causation, whereby disease-associated physiological changes alter the microbiota rather than result from it [196,251]. Furthermore, increasing evidence indicates that functional properties often differ between strains belonging to the same microbial species, highlighting the limitations of taxonomic analyses that do not capture strain-level variation [215,241,242]. Finally, although animal models provide essential mechanistic insights, important differences in gastrointestinal physiology, immune system organization, and microbiota composition limit the direct translation of experimental findings to humans [34,110,113].

Another key priority is the integration of mechanistic and systems biology approaches. By combining high-resolution metagenomics with host transcriptomics, metabolomics, and epigenomics, researchers can uncover causal pathways that connect microbial metabolites with immune effector mechanisms [109,174]. Such integrative strategies will refine our understanding of how dietary inputs, xenobiotics, and biologic therapies reprogram immune networks via the microbiome.

Ethical and sustainability considerations will increasingly shape experimental design. The refinement of in vitro gut simulators, microfluidic “gut-on-chip” systems, and organoid co-cultures can significantly reduce the dependence on animal models, aligning microbiome research with the 3Rs principle [111,231]. These systems also allow controlled testing of new biologic and IgY-based therapeutics, providing reproducible data under physiologically relevant conditions.

In the future, personalized microbiota modulation is expected to become a central strategy in preventive and therapeutic medicine. Artificial intelligence-driven modeling, coupled with real-time microbiome monitoring, could predict host responses to specific microbial or dietary interventions. The convergence of microbiome science with immunotherapy, nutrigenomics, and precision medicine will define the next generation of targeted immuno-microbiome therapies that promote long-term immune balance, metabolic resilience, and healthy aging [115,232,243].

In conclusion, the gastrointestinal tract represents a highly dynamic biological interface in which epithelial structures, microbial communities, and immune mechanisms continuously interact to maintain mucosal and systemic homeostasis. Across mammalian species, increasing evidence demonstrates that the gut microbiota actively shapes epithelial integrity, immune maturation, metabolic regulation, and inflammatory signaling through complex bidirectional communication networks. Microbial metabolites, epithelial sensing pathways, and host immune responses therefore function as an integrated regulatory system rather than isolated components. A deeper mechanistic understanding of these interactions, supported by multi-omics technologies, advanced in vitro models, and comparative translational research, will be essential for the development of targeted microbiome-based interventions in both human and veterinary medicine.

## Figures and Tables

**Table 1:** Comparative functional features of the gastrointestinal tract across humans and commonly used animal models in gut microbiome research.

Species	GIT Length	Fermentation Niche	Mucus	Gut-Associated Lymphoid Tissue (GALT)	Model Suitability
Human	~9 m	Colon	Dual layer	Peyer’s and lymphoid follicles	Human health context
Mouse/Rat	~1–2 m	Caecum	Single layer	Peyer’s and cecal patch	Immune/gnotobiotic studies [56,57,58]
Pig	~20 m	Colon, caecum	Dual layer	Multifollicular clusters	High translational relevance [59]
Zebrafish	~3–4 cm	No defined chamber	Single mucus layer	Diffuse gut-associated leukocytes (no organized GALT)	Transparent larvae, real-time imaging of host–microbe–immune interactions [49]
*Drosophila*	~2–3 mm	Crop and midgut	Peritrophic matrix	No GALT, innate immunity only	Genetic tractability, conserved innate immune signaling (Toll, IMD), high-throughput screening [60]

**Table 2:** Comparative features of the gut microbiome across representative mammalian species.

Host Group	Dominant Phyla	Predominant Microbial Traits	Distinctive Features and Translational Notes	References
Humans	*Firmicutes*, *Bacteroidetes*, *Actinobacteria*, *Verrucomicrobia*	Highly diverse community adapted to mixed diets	Highly diverse, diet-responsive community; benchmark for translational studies	[6,7,73,74]
Rodents (mice/rats)	*Firmicutes*, *Bacteroidetes*	Cecum-centered fermentation	Widely used mechanistic and gnotobiotic models	[34,35,37,50,58]
Pigs	*Firmicutes*, *Bacteroidetes*, *Spirochaetes*	Composition and ecology closely resembling humans	High translational relevance for microbiome studies	[8,59,62]
Ruminants (cattle, sheep, goats)	*Firmicutes*, *Bacteroidetes*, *Fibrobacteres*, *Archaea*	Specialized fiber-degrading microbiota associated with foregut fermentation	Model for host–microbe coadaptation and fiber digestion	[62]
Carnivores (dogs, cats)	*Firmicutes*, *Fusobacteria*	Protein-adapted microbiota with lower fermentative capacity	Distinct dietary specialization and reduced diversity	[62]

Note: Although the relative abundance of microbial taxa varies among mammalian hosts, *Firmicutes* and *Bacteroidetes* constitute dominant components of the gut microbiota in most species [62].

**Table 3:** Major mucosal immune connections linking gastrointestinal, respiratory and urogenital tissues.

Axis	Anatomical Connections	Principal Immune Mechanisms	Physiological Influence	References
Gut–lung axis	Gut and respiratory mucosa	Lymphocyte recirculation, IgA, cytokines	Pulmonary immune regulation	[91,92,93,94,95]
Gut–urogenital axis	Gut and urogenital mucosa	Lymphocyte homing, IgA-mediated protection	Contributions to the urogenital immune balance	[96,97,98,99]
Gut–oral axis	Gut and oral mucosa	Antibody responses and immune cell trafficking	Regulation of oral and gastrointestinal microbial communities	[100,101,102,103,104]

**Table 4:** Major microbiota-derived metabolites involved in host immune regulation.

Metabolite Class	Main Producing Taxa/Pathways	Major Host Receptor(s)/Targets	Principal Immune Effects	References
Secondary BAs (DCA, LCA, isoallo-LCA, 3-oxo-LCA)	*Clostridium scindens*, *C. hylemonae*; *Firmicutes* carrying the *bai* operon; BSH-positive bacteria mediating deconjugation and epimerization	FXR, TGR5 (GPBAR1); direct transcriptional modulation in T cells	Suppression of Th17 differentiation (3-oxo-LCA); induction of Tregs (isoallo-LCA); modulation of mucosal immune tolerance and epithelial homeostasis	[140,141,143,144,145]
Indole derivatives (IAA, ILA, IPA, oxindoles)	*Lactobacillus* spp. (especially ILA); secondary indole producers including *Clostridium* spp.; microbial tryptophan metabolism	AhR; type I IFN–associated signaling	Enhanced epithelial barrier integrity; IL-22 induction; anti-inflammatory signaling; protection from mucosal immune injury; microbial cross-feeding amplification	[149,150,151,152,154]
SCFA (acetate, propionate, butyrate)	Fiber-fermenting anaerobes; acetate via Wood–Ljungdahl pathway, propionate via succinate/acrylate pathways, butyrate via acetyl-CoA condensation	FFAR2 (GPR43), FFAR3 (GPR41); HDAC inhibition	Promotion of Treg differentiation; macrophage polarization toward anti-inflammatory phenotypes; suppression of pro-inflammatory cytokines; GLP-1 and PYY regulation	[108,155,156,160,161]
Polysaccharide A	*Bacteroides fragilis*	TLR2-dependent immune signaling	Expansion of IL-10–producing Tregs; maintenance of immune tolerance	[85]
Vitamin B2 metabolites	Riboflavin-synthesizing commensals	MR1 → MAIT cell activation	MAIT cell maturation and mucosal immune surveillance	[89,90,170,171]
Microbial LPS/endotoxins	Expansion of Gram-negative facultative anaerobes (e.g., *Enterobacteriaceae*)	TLR4, NF-κB pathways	Chronic low-grade inflammatory activation; endotoxemia	[77,173]

**Table 5:** Microbiota-targeted Therapeutic Strategies and Translational Status.

Strategy	Representative Examples	Primary Indication(s)	Evidence Type	Mechanism of Action	Clinical/Regulatory Status	Limitations and Safety Considerations
Probiotics	*Lactobacillus rhamnosus GG*, *Bifidobacterium longum*	IBD; antibiotic-associated dysbiosis; functional GI disorders	Preclinical studies, RCTs, meta-analyses	Barrier reinforcement. increased mucosal IgA; attenuation of inflammatory signaling	Available as dietary supplements; strain-specific efficacy;	Heterogeneous clinical outcomes; limited colonization; strain-dependent effects [215]
Prebiotics	Inulin; FOS; resistant starch	IBD, metabolic disorders	Preclinical studies and clinical trials	Production of SCFA-producing taxa; promotion of Treg-mediated immune tolerance	Nutritional immunomodulation in IBD and metabolic inflammation;	Interindividual variability; GI tolerance at higher doses; growing mechanistic support [216,217,218,219,220]
FMT (established use)	Donor-derived whole microbiota transfer	Recurrent *Clostridioides difficile* infection	Multiple RCTs and guideline-supported clinical evidence	Restoration of microbial ecosystem structure and function	Established clinical therapy for recurrent CDI in several jurisdictions,	Donor screening requirements; pathogen transmission risk; regulatory variability [221,222,223,224]
FMT (investigational use)	Whole microbiota transfer; defined microbiota consortia	IBD, metabolic syndrome, HSCT-related complications	Early-phase clinical studies	Modulation of microbiota composition and immune responses	Investigational	Variable engraftment; inconsistent efficacy; lack of standardization [62,82,205,206,207,208,209,210]
Personalized FMT	Age-matched donors; tailored consortia	Pediatric and immunocompromised patients	Proof-of-concept and early clinical studies	Improved donor–recipient compatibility and engraftment	Experimental	Limited clinical validation; logistical complexity [225,226]
Synbiotics	*Lactobacillus*—inulin; *Bifidobacterium*–GOS combinations	Dysbiosis-associated disorders	Preclinical and clinical studies	Additive or potentially synergistic effects reported on early studied for colonization and metabolite production with individual components	Investigational/adjunctive approach	Benefits are formulation-dependent and not consistently demonstrated [182,227]
Next-generation probiotics and postbiotics	*Faecalibacterium prausnitzii*; *Akkermansia muciniphila*; defined metabolites	Inflammatory and metabolic disorders	Mainly pre-clinical and early clinical studies	Targeted immunomodulation of the host and metabolic pathways	Translational research phase	Manufacturing, stability, regulatory and safety challenges remain [228]

## Data Availability

No new data were created or analyzed in this study.

## Abbreviations

The following abbreviations are used in this manuscript:

3Rs	Replacement, Reduction, and Refinement (ethical principle for animal use)
AhR	Aryl hydrocarbon receptor
AMP	Antimicrobial peptide
APC	Antigen-presenting cell
BA	Bile acid
BSH	Bile salt hydrolase
CA	Cholic acid
CD	Crohn’s disease
CDCA	Chenodeoxycholic acid
DC	Dendritic cell
DCA	Deoxycholic acid
DKD	Diabetic kidney disease
EECs	Enteroendocrine cells
F/B ratio	Firmicutes/Bacteroidetes ratio
FMT	Fecal microbiota transplantation
FXR	Farnesoid X receptor
GALT	Gut-associated lymphoid tissue
GI	Gastrointestinal
GIT	Gastrointestinal tract
GPCR	G-protein-coupled receptor
HDAC	Histone deacetylase
IAA	indole-3-acetic acid
ILA	indole-3-lactic acid
IPA	indole-3-propionic acid
IEL	Intraepithelial lymphocyte
IgA	Immunoglobulin A
IgY	Immunoglobulin Y (Avian antibody)
ILC	Innate lymphoid cell
IL	Interleukin
IBD	Inflammatory bowel disease
JAK	Janus kinase
LCA	Lithocholic acid
MAIT	Mucosal-associated invariant T cell
MAMP	Microbe-associated molecular pattern
MR1	Major histocompatibility complex class I-related molecule
MyD88	Myeloid differentiation primary response protein 88
NF-κB	Nuclear factor kappa-light-chain-enhancer of activated B cells
NGS	Next generation sequencing
NLR	NOD-like receptor
NOD2	Nucleotide-binding oligomerization domain-containing protein 2
PAMP	Pathogen-associated molecular pattern
pIgR	Polymeric immunoglobulin receptor
PRR	Pattern-recognition receptor
PSA	Polysaccharide A
RA	Retinoic acid
RORγt	Retinoic acid related orphan receptor γt
SCFA	Short-chain fatty acid
sIgA	Secretory immunoglobulin A
TGF-β	Transforming growth factor beta
Th	Helper T cell
TLR	Toll-like receptor
TNF	Tumor necrosis factor
Treg	Regulatory T cell
UC	Ulcerative colitis
Wnt	Wingless/Integrated signaling pathway
